# Ruptured Noncommunicating Rudimentary Horn Pregnancy at 19 Weeks with Previous Cesarean Delivery: A Case Report

**DOI:** 10.1155/2012/308476

**Published:** 2012-10-18

**Authors:** Sita Thakur, Ajay Sood, Chanderdeep Sharma

**Affiliations:** Dr. Rajendra Prasad Government Medical College, Kangra at Tanda (HP) 176001, India

## Abstract

Unicornuate uterus with noncommunicating rudimentary horn occurs due to incomplete fusion of mullerian ducts. Pregnancy in this horn is a rare phenomenon usually resulting in rupture during second trimester of pregnancy. Prerupture diagnosis of pregnancy in rudimentary horn with ultrasonography is technically difficult, with sensitivity of 30%. We report a case of ruptured non-communicating rudimentary horn at 19 weeks in a woman with previous Cesarean delivery. She had a routine malformation scan in which diagnosis was missed. Later she presented to emergency in shock, with massive hemoperitoneum and ruptured horn. So a high index of suspicion is required to save this catastrophic event and associated maternal morbidity and mortality. In our opinion, routine excision of rudimentary horn should be undertaken during nonpregnant state laparoscopically. However, those women who refuse should be adequately counseled regarding potential complications and if pregnancy occurs in rudimentary horn, first trimester laparoscopic excision should be done.

## 1. Introduction

Unicornuate uterus with a rudimentary horn is a rare mullerian anomaly that has a high incidence of obstetric complications that include ectopic pregnancy in the rudimentary horn [1]. Pregnancy in noncommunicating rudimentary horn is possible by trans-peritoneal migration of sperm or fertilized ovum. It occurs in approximately 1 of every 76,000 pregnancies. The risk of uterine rupture is 50–90%, with most ruptures (approximately 80%) occurring by the end of the second trimester [1, 2]. We report a case of second trimester rupture of noncommunicating rudimentary horn which was missed on routine malformation scan.

## 2. Case Report

**Figure 1:**
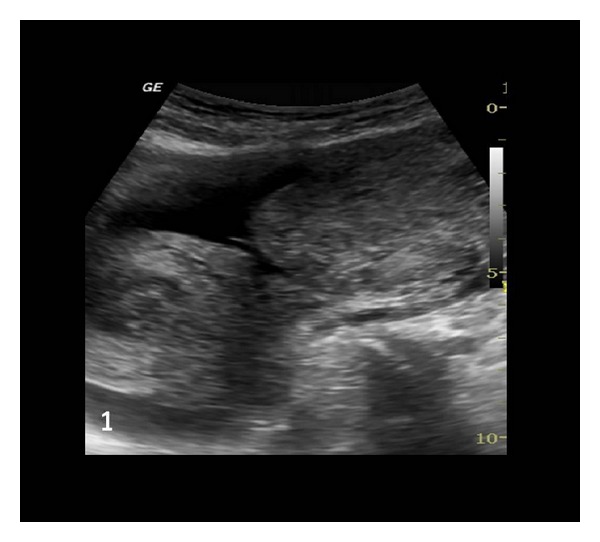
Unicornuate uterus with noncommunicating ruptured rudimentary horn (both are empty, no products of conception seen).

**Figure 2:**
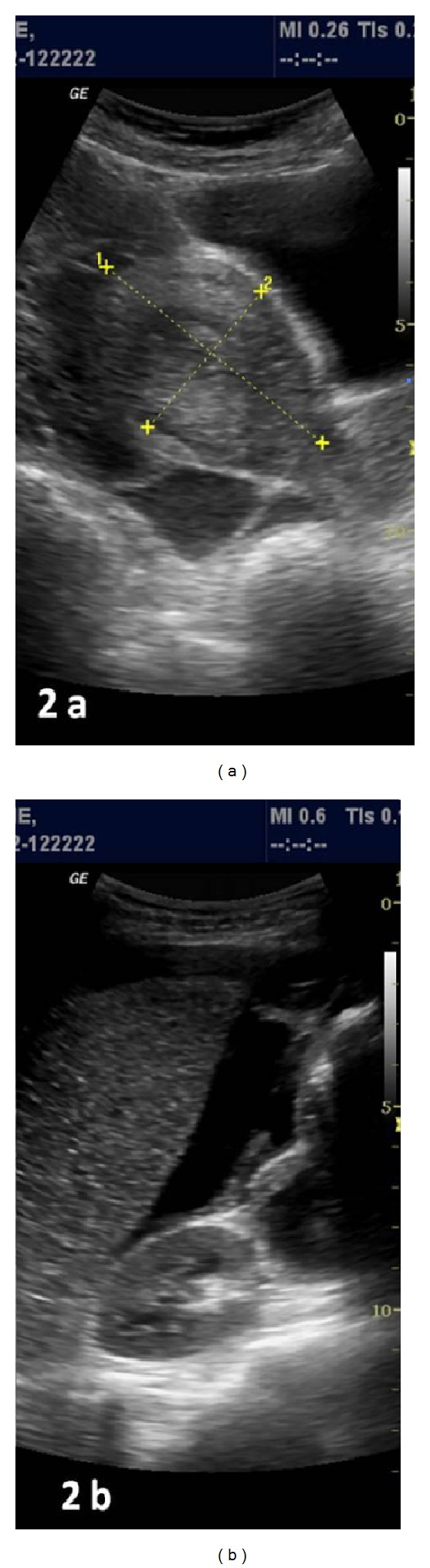
(a) Rudimentary horn with rupture of posterior wall and pelvic collection; (b) moderate free fluid in Morrison's pouch.

**Figure 3:**
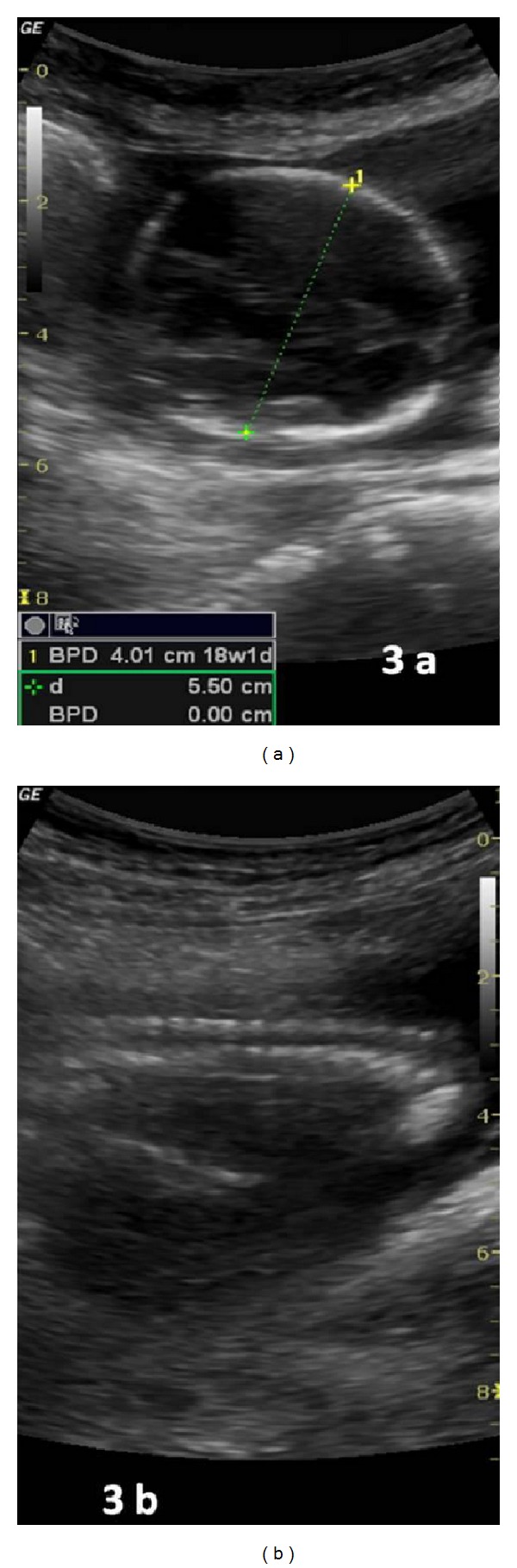
(a) Fetal head lying in peritoneal cavity; (b) fetal spine lying in peritoneal cavity.

**Figure 4:**
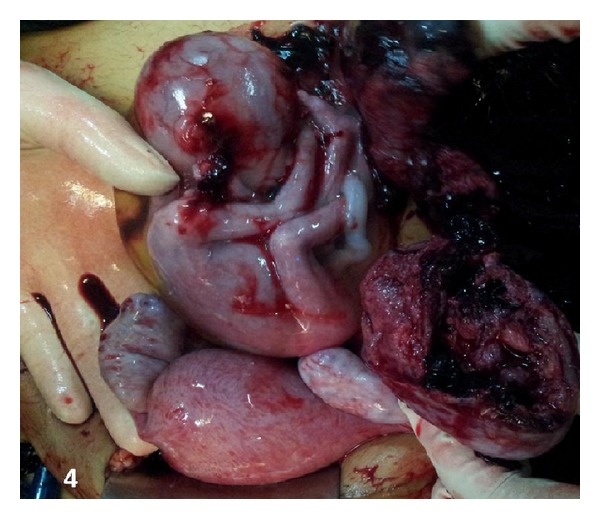
Unicornuate uterus with rupture of pregnant noncommunicating rudimentary horn, with fetus lying outside the uterus (intraoperative).

**Figure 5:**
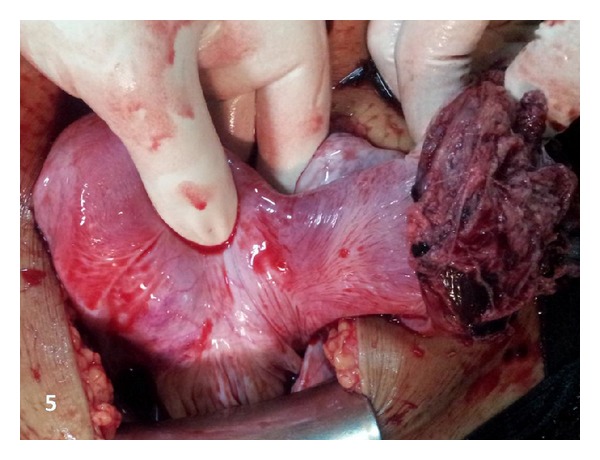
Unicornuate uterus with rupture of rudimentary horn, previous cesarean scar healthy.

A 26-year-old woman gravida 1, para 1 came to emergency with severe lower pain abdomen and vomiting since last two hours. She was 18 weeks pregnant and her routine malformation scan done one week back was normal. Her previous delivery was by Cesarean section for transverse lie. She denied history of dysmenorrhoea or of pain earlier in previous pregnancy. The patient had no significant medical or surgical history. No records of previous cesarean section were available. On examination patient was in hypovolemic shock with severe pallor, hypotension, and tachycardia. The abdomen was tense and symphysiofundal height was 24 weeks. Her bowel sounds were normal. On pelvic examination cervix and vagina were healthy, there was no bleeding through os, and size of uterus could not be made out due to intense guarding. Per rectal examination was within normal limits. Immediately two large bore intravenous cannulas were inserted, one liter of fluid was rushed, patient was catheterized (she passed 50 mL of clear urine), and urgent investigations and cross match was sent for four units of blood. Her hemoglobin was 4.6 g%, one unit blood was rushed, and after stabilization urgent emergency ultrasound was done. Her uterus was found to be empty, with a hyperechoic shadow adjacent to it. There was marked free fluid in abdomen. Immediately consultant review was sought, which revealed unicornuate uterus with noncommunicating rudimentary horn (Figure 1). The rudimentary horn was found to be ruptured on posterolateral wall (Figure 2(a)) with moderate free fluid in peritoneal cavity (Figure 2(b)). A dead fetus was found floating in the abdominal cavity (Figures 3(a) and 3(b)). The patient was taken for explorative laparotomy. Intraoperatively a unicornuate uterus with rupture of noncommunicating rudimentary horn was confirmed and a dead fetus was found in peritoneal cavity with four liters of hemoperitoneum (Figures 4 and 5). There was no endometrial cavity in the noncommunicating horn. Both the ovaries and tubes were normal (Figures 4 and 5). Previous cesarean scar was healthy (Figure 5). Placenta was found separated in abdominal cavity. Excision of rudimentary horn, ipsilateral salpingectomy, and peritoneal toileting was done. Patient received five units of blood transfusion. She had an uneventful recovery and was discharged on day 7 post operative with an advice for hysterosalpingogram and intravenous pyelogram 6 weeks later.

## 3. Discussion

Mariceau and Vassal published the first description of a rudimentary horn pregnancy in 1669, and 600 cases have since been described [3]. Pregnancies occur in both communicating and noncommunicating horns in proportion to their relative incidence and are equally likely to rupture [2]. Neonatal mortality is very high as most cases are emergency laparotomies after uterine rupture at premature gestational age [4, 5]. Maternal mortality is low (0.5%) [2] but morbidity is very high in view of massive blood loss and morbidly adherent placentation [2, 4, 5].

The prerupture diagnosis of pregnancy in rudimentary horn has drastically reduced maternal mortality [3]. But the sensitivity of ultrasound to detect prerupture rudimentary horn pregnancy is very poor (30%) [4, 5], probably because of rarity of the diagnosis and nonfamiliarity of the radiologists about this potentially lethal condition. Early diagnosis before rupture can be managed laparoscopically by immediate excision of the horn, pregnancy, and the ipsilateral fallopian tube [5].

 Tsafrir et al. proposed the following criteria for ultrasonographic diagnosis: (1) a pseudo pattern of an asymmetrical bicornuate uterus, (2) absent visual continuity tissue surrounding the gestation sac and the uterine cervix, and (3) the presence of myometrial tissue surrounding the gestation sac [6]. In any doubtful case three-dimensional ultrasound or magnetic resonance imaging should be done to avoid the potential complications.

This case highlights the fact that despite having risk factor for suspected uterine anomaly, that is, previous cesarean section for fetal malpresentation, this patient was missed on routine malformation scan one week prior to the catastrophic rupture of the rudimentary horn. Her previous operative records were not reviewed, which when subsequently reviewed clearly stated the presence of unicornuate uterus with noncommunicating rudimentary horn. This woman was also not warned regarding potential complications. Hence this case is being reported to familiarize the radiologists regarding this rare but potentially lethal presentation which if diagnosed safely in prerupture state can be managed laparoscopically without the associated sequelae of rupture uterus. Three-dimensional ultrasound imaging and MRI are useful tools in the improvement of diagnostic accuracy, guiding both counseling and surgical planning [3]. 

This case further raises the question of whether routine excision of rudimentary horn be undertaken in women with unicornuate uterus as a prophylaxis to prevent such catastrophes.

 A further evaluation of timing of such a surgery is required in a case series, which seems highly unlikely considering the rarity of the condition. In our opinion this decision should be extrapolated from isolated case reports only and routine laparoscopic excision of rudimentary horn with ipsilateral fallopian tube should be offered to these women and those refusing should be adequately counseled regarding the potential complications and if pregnancy occurs in rudimentary horn first trimester laparoscopic excision should be done.
